# Validation of an Arabic version of the short form of the health literacy in dentistry scale: a cross-sectional study

**DOI:** 10.1186/s12903-024-04303-9

**Published:** 2024-05-29

**Authors:** Muneera Essa Alzeer, AlBandary Hassan AlJameel, Kasper Rosing, Esben Boeskov Øzhayat

**Affiliations:** 1 Department of Odontology, University of Copenhagen, Copenhagen, Denmark; 2 Department of Dental Health, Colleges of Applied Medical Sciences, King Saud University, Riyadh, Kingdom of Saudi Arabia; 3 Department of Periodontics and Community Dentistry, College of Dentistry, King Saud University, Riyadh, Kingdom of Saudi Arabia

**Keywords:** Oral health, Health literacy, Oral health literacy, HeLD-14, Saudi Arabia, Validity, School students

## Abstract

**Background:**

The Arabic version of the short form of the Health Literacy in Dentistry scale (HeLD) had not yet been developed in previously published studies. This study aims to test the reliability and validity of an Arabic version of the short form of the HeLD questionnaire.

**Methods:**

The short version of HeLD was translated into Arabic and the psychometric properties were evaluated in a sample of 1,889 female students in their first year of secondary school. Test–retest reliability was assessed using the intra-class correlation coefficient (ICC), and internal consistency reliability was assessed using Cronbach’s alpha. Content validity was investigated by creating a correlation matrix between the individual items of the HeLD-14, and criterion validity was determined using Pearson’s correlation between the HeLD-14 score and an overall oral health rating. Sensitivity analysis was assessed by testing the associations of the HeLD-14 score with oral health-related behaviours and residential area.

**Results:**

The Arabic version of HeLD-14 (A-HeLD-14) had acceptable ICC (0.54) and excellent internal consistency (Cronbach’s alpha: 0.92). The correlations between the items of the A-HeLD-14 varied from 0.3 to 0.9. The A-HeLD-14 showed a statistically significant correlation with the overall oral health rating (*r* = 0.37, *p* < 0.001). The median A-HeLD-14 score was significantly higher in participants who brushed their teeth frequently (51.31), visited the dentist regularly (52.00), consumed fresh fruit frequently (51) and consumed soda or energy drinks infrequently (51.00) than participants who brushed their teeth infrequently (41.50), visited the dentist irregularly (49.00), consumed fresh fruit infrequently (47) and consumed soda or energy drinks frequently (48.00).

**Conclusion:**

The A-HeLD-14 instrument demonstrates sufficient validity, reliability, and sensitivity for measuring oral health literacy among the Arabic-speaking population.

## Background

Oral diseases are a major health burden worldwide [1], affecting about half of the world population. This is also true in the Middle East, where the prevalence of the most common oral diseases has increased significantly [2]. Over 40 million children in Arabic-speaking countries have untreated carious lesions in deciduous teeth, and an estimated 37 million people in these countries have unmet treatment needs in the permanent dentation [3]. Thus, oral diseases pose a significant burden on quality of life and healthcare systems in the Middle East [2].

The primary cause of poor oral health in the Middle East is considered to be poor oral health behaviours, including insufficient oral hygiene practices, high sugary diet consumption, and infrequent use of the dental care system [2, 4]. This warrants increased focus on heath literacy, which is the “degree to which individuals have the capacity to obtain, process and understand the basic health information and services needed to make appropriate health decisions” [5, 6]. Health literacy is a strong predictor of health, health behaviour, and health outcomes [7, 8] and is one of the key strategies for promoting health according to the World Health Organisation (WHO) [9].

Specifically focusing on oral health literacy (OHL) is relevant, as OHL is vital for achieving good oral health [10]. OHL interventions have been found to reduce oral health disparities and improve oral health [11], which is why the number of studies on OHL has increased in the dental literature over the last decade [12].

To reap the benefits of this increased focus on OHL requires valid and reliable measures for assessing OHL. So far, the most widely used and available OHL measure for Arabic-speaking populations has been the Rapid Estimate of Adult Literacy in Dentistry (REALD) [13]. However, REALD does not include any specific OHL domains and therefore has limited use when aiming to improve oral health through OHL interventions. The Health Literacy in Dentistry (HeLD) scale is an OHL instrument [14] that could be used to pinpoint OHL from different domains as it represents seven conceptual OHL domains: access, understanding, support, utilisation, economic barriers, receptivity, and communication [15]. The HeLD originally comprised 29 items, but a short version of 14 items (HeLD-14) has been developed, tested, and found to be valid and reliable [14]. The main advantage of the short version of HeLD is its ease of use in epidemiological studies. The HeLD-14 has been culturally adapted and tested in different languages and cultures including Brazilian [16], Chinese [17], Malay [18], and Persian versions [19]. A newly published study in the Kingdom of Saudi Arabia (KSA) has developed an Arabic version of the HeLD-14 [20]. The investigators aimed to assess the OHL among schoolteachers. However, the study did not present much information on the validity and reliability of the instrument [20]. A valid and reliable version of an Arabic version of the HeLD-14 is thus needed and relevant to introduce.

Mothers in the KSA are considered the cornerstone of the family, playing a crucial role in childcare and various family responsibilities [21]. Their influence extends to shaping the behaviours of their family members, especially the children [21], ultimately impacting their oral health-related behaviours. Further, a strong relationship has been found between maternal OHL, oral health-related knowledge, attitude and practices, and children’s oral health status [22–24]. It is thus imperative to evaluate the OHL of women, as this can facilitate the implementation of effective intervention programmes that will not only enhance women’s oral health, but also have a positive impact on their children’s oral health. Assessing OHL in an adolescent population is essential as this is a crucial point in life where health behaviours are manifested [25]. The resulting OHL targets and interventions could promote higher levels of OHL among tomorrow’s adults, thereby preventing oral health problems in both themselves and future generations.

We therefore aimed to adapt and test the validity and reliability of an Arabic version of the HeLD-14 (A-HeLD-14) in a young female population. The measure will not only enable the assessment of OHL levels in Arabic-speaking countries, but also pinpoint the main barriers to improving OHL. This could be useful for decision makers and oral healthcare workers when developing and implementing effective oral health initiatives. Further, the measure could be highly useful in epidemiological studies in Arabic-speaking countries to monitor oral health or evaluate new oral health policies. The null hypothesis for this study was that the A-HeLD-14 is not a valid and reliable instrument, and the alternative hypothesis was that the A-HeLD-14 is a valid and reliable instrument for assessing OHL in an Arabic-speaking population.

## Methods

### Study design

Data were collected from January 2022 to October 2022 as a part of a larger cross-sectional study using a questionnaire to assess the oral health status and behaviours of 10th grade female students in the Eastern Province of the KSA.

The study entailed two phases. The first phase involved translating the questionnaire from English into Arabic and the second phase entailed testing the psychometric properties of the A-HeLD-14.

### Ethical approval

Participants were anonymised, and their data kept confidential. Prior to data collection, the research protocol was approved by the Institutional Review Board at King Saud University Medical City in the KSA (approval letter reference number: 21/0309/IRB).

### Setting and participants

**Fig. 1:**
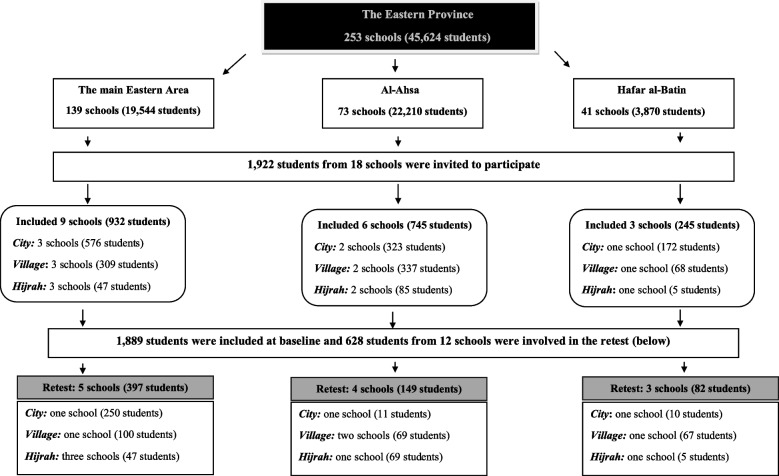
The distribution of schools in clusters

The study took place in the Eastern Province in the KSA, which comprises three main governorates: the main Eastern area; Al-Ahsa; and Hafar al-Batin. These areas formed the clusters for this study. The largest governorate is the main Eastern area (population: 5,148,598) [26], followed by Al-Ahsa (population: 1.3 million) and Hafar al-Batin (population: 365,000). Each governorate is divided into urban (cities) and rural areas (villages and hijrahs) constituting the three strata in this study. A *hijrah* in the KSA is a tribal society that is usually located in a desert area. Hijrahs are smaller than villages and have fewer living facilities. Each stratum has public high schools for females, and these were the units where the study was performed (Fig. 1)*.*

The study sample size was calculated based on the expected level of OHL in the population and the possibility of detecting a significant difference between the groups described in the sensitivity analysis. The mean OHL was set to 46 in the group hypothesised to have higher OHL and to 42 in the group hypothesised to have lower OHL. The high means are due to the study population, who we expected to have a generally high OHL. With a power of 90% and a significance level set to 0.05, a total of 190 participants per group were needed to obtain significant results.

The schools were randomly chosen using Stratified Random Sampling Technique via the Random Choice Generator [27]. We selected three schools from each stratum of the main Eastern area. Two schools were selected from each stratum of Al-Ahsa, and one school was selected from each stratum of Hafar al-Batin. The number of schools in each cluster was based on the population and size of the geographic area. All 10th grade students from the selected schools (*n* = 18) were invited to participate, and informed consent for participation was obtained from their parents.

### Questionnaire and variables

#### Oral health literacy

OHL was measured using the HeLD-14 questionnaire (Table 1), which represents seven conceptual domains: communication, access, receptivity, understanding, utilisation, support, and economic barriers [15]. Each domain has a set of questions (items) and each item is scored on a 5-point Likert scale ranging from 0 (“unable to do”) to 4 (“without any difficulty”). A higher score indicates a higher level of oral health literacy. The total HeLD-14 score is the unweighted summation of the item scores [14], and the final score for the HeLD-14 thus ranges from 0 to 56.

**Table 1:** The domains and the associated items for HeLD-14

Domain	Items
Communication	*1. Are you able to* look for a second opinion?
	*2. Are you able to* use information?
Access	*3. Do you know* how to get dentist’s appointment?
	*4. Do you know* what to do to get a dental appointment?
Receptivity	*5. Are you able to* pay attention to dental health needs?
	*6. Are you able to* make time for things that are good for dental health?
Understanding	*7. Are you able to* fill in dental forms?
	*8. Are you able to* read dental information brochures?
Utilisation	*9. Are you able to* carry out dental instructions?
	*10. Are you able to* use the dentist’s advice?
Support	*11. Are you able to* take support to a dental appointment?
	*12. Are you able to* ask for support at dental appointment?
Economic barriers	*13. Are you able to* pay to see a dentist?
	*14. Are you able to* pay for dental medication?

#### Oral health-related behaviours

The following oral health-related behaviours were reported: frequency of toothbrushing [28, 29], dietary habits [29], and pattern of dental attendance [28, 29]. Answer categories for the frequency of toothbrushing were: never, rarely, several times a month, several times a week, once a day usually in the morning, once a day usually before bed, and several times a day. Dietary habits concerned the consumption frequency of (i) fresh fruit and (ii) soda or energy drinks and the answer categories were: several times daily, every day, several times a week, once weekly, several times a month, and never. The answer categories for the pattern of dental attendance were regularly, approximately once a year; regularly, approximately twice a year or even more often; when having a toothache or other acute problems; and do not visit the dentist.

For the statistical analysis, the original answer categories for the oral health-related behaviours were dichotomised. Toothbrushing was deemed frequent if performed once a day or several times a day and infrequent if performed several times a week or month, rarely, or never. Dietary habits were designated as frequent if the dietary items were consumed several times daily, every day, or several times a week and infrequent if they were consumed once weekly, several times a month, or never. Dental attendance was categorised as regular if the participant reported visiting a dentist once or more a year and irregular if the visits were related to a toothache or other acute problems occurring, or never visited a dentist.

#### Overall rating of oral health

Overall rating of oral health was assessed using the question: *In your opinion, how would you rate your oral or dental health?* [30] The answer categories were: very bad; bad; fair; good; and very good.

#### Cross-cultural adaptation process

The full questionnaire including the HeLD-14, originally in English, was translated into Arabic by two native Arabic-speaking professional translators. Next, the combined Arabic version of the questionnaire was translated back into English by two other professional translators; this version was reviewed by two of the authors (A.A. and M.A.) and compared with the original version to discover any discrepancies [31]. The level of linguistic homogeneity was deemed acceptable and the level of translation and cultural adaptation sufficient. The translated version showed no major discrepancies when compared with the original version of the questionnaire.

Operational equivalence suggests the practicality of using a questionnaire format, instructions, measurement technique, and method of administration in different target populations that are similar to those previously employed in the original setting [32]. In this context, we conducted a pilot test in two schools located in the Eastern Province of the KSA that represented various socio-economic backgrounds. The purpose was to assess the feasibility of conducting the study. This included addressing logistics such as school access and finding the most effective methods of distributing the questionnaire, delivering virtual instructions, enhancing participant engagement, and presenting the questionnaire in a clear manner.

### Data collection

Participants completed the questionnaire electronically using the Google Forms software provided to the school principals via a link posted to the students through the schools’ official portals. Each participant was assigned a unique code, allowing us to match the questionnaires with the respective informed consent forms. For test–retest purposes, 12 out of the 18 schools were invited to fill in the A-HeLD-14 questionnaire a second time from 2 weeks to 1 month after completion of the initial data collection (Fig. 1). The selection of schools for test–retest was based on the degree of compliance and the response rate from the schools in the urban and rural areas of all three clusters.

### Psychometric testing

The psychometric testing and corresponding analyses were based on theoretically described approaches [33]. We thus used content and criterion validity in addition to reliability tests and sensitivity analysis. All tests and analyses are described in the following sections.

#### Test–retest reliability

Test–retest reliability, sometimes referred to as stability or reproducibility, concerns the consistency of scores across two separate measurements over time [34]. Intra-class correlation coefficient (ICC) was used to assess test–retest reliability. An ICC below 0.40 indicates a poor level of consistency, 0.40–0.59 indicates a fair level of consistency, 0.60–0.74 indicates a good level of consistency, and 0.75–1.00 indicates an excellent level of consistency [35]. According to Kennedy (2022), the recommended sample size for achieving high reliability for test–retest is at least 100 participants [36]. In this study, 628 students were invited to take part in the test–retest reliability study, and 615 students were included.

#### Internal consistency reliability

Internal consistency reliability is based on item-to-item correlations in multi-item scales [37]. Cronbach’s alpha coefficient was used to measure internal consistency. A Cronbach’s alpha coefficient of 0.70 or higher is considered satisfactory for showing the internal consistency reliability of the research instrument [38].

#### Content validity

This measures the degree to which elements of an assessment instrument are relevant to and representative of the targeted construct for a particular assessment purpose [39]. The content validity of the English version of the HeLD-14 has been investigated in other studies [14, 16], and since the Arabic version was a translation of this, we accepted the relevance of the questions without further investigation. The content was further reviewed by author A.A. and a high-standards committee from Saudi Ministry of Education for any obvious flaws or offensive phrases. Content validity was further investigated by creating a correlation matrix between the individual items of the A-HeLD-14. If the correlation between any two aspects was above 0.5, a possible overlap between the aspects was considered.

#### Criterion validity

Criterion validity reflects how accurately a test measures the outcome [40]. This can be done by comparing the new measure to a gold standard. The A-HeLD-14 score in this study was compared to the overall rating of oral health using Pearson’s correlation coefficient *r*. A correlation of 0.4 < *r* ≤ 0.6 indicates a moderate correlation, with an acceptable level of* r* being 0.5 and above [41].

#### Sensitivity analysis

Sensitivity was assessed by exploring the associations of the A-HelD-14 score with the included oral health-related behaviour variables and the strata. We hypothesised that the A-HeLD-14 score would be higher for participants with regular toothbrushing, regular dental attendance, frequent fresh fruit consumption, and infrequent soda and energy drink consumption and who live in a city compared to participants with irregular toothbrushing, irregular dental attendance, infrequent fresh fruit consumption, and frequent soda or energy drink consumption and who live in a village or hijrah. Because the A-HeLD-14 scores were not normally distributed, the Mann–Whitney U test, the Kruskal–Wallis H test, and Receiver Operating Characteristic (ROC) curves were used to assess the associations. The cut-offs for the Area Under Curve (AUC) score yielded from the ROC curves were 0.5 = no discrimination; 0.5–0.7 = poor discrimination; 0.7–0.8 = acceptable discrimination; 0.8–0.9 = excellent discrimination; and > 0.9 = outstanding discrimination [42].

### Statistics

Data from the questionnaires were cleaned, and IBM SPSS software version 28 was used for data analysis. Descriptive statistics were used to determine the values of the median and Interquartile Range (IQR) of the A-HeLD-14 score. A statistical significance level of 0.05 was applied.

## Results

### Participants

Of the 1,922 students invited to participate, 1,889 (98.3%) students were included. The main reasons for exclusion were missing informed consent (*n* = 1), negative informed consent (*n* = 1), the student transferred to another school during the data collection period (*n* = 11), the student declined participation in the study (*n* = 3), and technically missing questionnaire (*n* = 17). Of the 628 students invited to complete the questionnaire for the A-HeLD-14 retest, 615 (97.9%) were included. Thirteen students were excluded due to technically missing questionnaire or missing informed consent. The selection of schools in the A-HeLD-14 retest study was based on the school’s response rate in the original study and the school’s level of compliance, meaning the cooperation of the principal, teaching and administrative staff, and students and their willingness to participate. Thus, of the 18 schools, 12 schools were included in the A-HeLD-14 retest study. Table 2 shows the distribution of participants for each variable, based on the original and the new categories.

**Table 2:** The distribution of participants according to clusters, strata, and oral health-related behaviours (*n* = 1889)

Distribution of the variables				
B Variable	**Original categories**	**Distribution** ***f* (%)**	**Collapsed categories**	**Distribution** ***f* (%)**
Cluster	Main Eastern area	920 (48.7)	NA	NA
	Al-Ahsa	732 (38.8)		
	Hafar al-Batin	237 (12.5)		
Stratum	City	1349 (71.4)	NA	NA
	Village	401 (21.2)		
	Hijrah	139 (7.4)		
Toothbrushing	Never	78 (4.1)	Infrequent	412 (21.8)
	Rarely	107 (5.7)	Infrequent	
	Several times a month	80 (4.2)	Infrequent	
	Several times a week	147 (7.8)	Infrequent	
	Once a day (morning)	238 (12.6)	Frequent	1477 (78.2)
	Once a day (evening)	201 (10.6)	Frequent	
	Several times a day	1038 (54.9)	Frequent	
Dental attendance	Regularly, twice a year or more often	313 (16.6)	Regular	550 (29.1)
	Regularly, approximately once a year	237 (12.5)	Regular	
	For toothache or other acute problems	1094 (57.9)	Irregular	1339 (70.9)
	I do not visit the dentist	245 (13.0)	Irregular	
Fresh fruit	Several times daily	505 (26.7)	Frequent	1371 (72.6)
	Every day	358 (19.0)	Frequent	
	Several times a week	508 (26.9)	Frequent	
	Once weekly	194 (10.3)	Infrequent	518 (27.4)
	Several times a month	259 (13.7)	Infrequent	
	Never	65 (3.4)	Infrequent	
Soda or energy drink	Several times daily	340 (18.0)	Frequent	1005 (53.2)
	Every day	281 (14.9)	Frequent	
	Several times a week	384 (20.3)	Frequent	
	Once weekly	311 (16.5)	Infrequent	884 (46.8**)**
	Several times a month	297 (15.7)	Infrequent	
	Never	276 (14.6)	Infrequent	

Most participants were living in the main Eastern area and in cities. Regarding the frequency of toothbrushing, 78.2% of the study sample brushed their teeth frequently and most brushed their teeth several times a day. Most participants visited the dentist only when they had a toothache or other acute problems. A minority of participants never visited dental clinics (13.0%). Regarding diet, most participants frequently consumed fresh fruit (72.6%), and about half consumed soda or energy drinks frequently (53.2%).

### Psychometric testing

#### Test–retest reliability

The ICC for the total A-HeLD-14 score in the test and retest was 0.54 (*n* = 615), indicating a fair consistency level between test and retest scores.

#### Internal consistency reliability

The Cronbach’s alpha was 0.92, indicating excellent internal consistency between the items.

#### Content validity

The correlation matrix shows correlations between the items from 0.3 to 0.9. The correlation is equal to or higher than 0.5 between items 1 and 2, items 3 and 4, items 5 and 6, items 5 and 9, items 5 and 10, and items 6 and 9, items 9 and 10, items 11 and 12, and items 13 and 14. The high and moderate correlation between the items of A-HeLD-14 occurs mostly between items located in the same domain (Table 3).

**Table 3:** The correlations between the individual A-HeLD-14 items. *n* = 1889

Correlation matrix														
	HeLD1	HeLD2	HeLD3	HeLD4	HeLD5	HeLD6	HeLD7	HeLD8	HeLD9	HeLD10	HeLD11	HeLD12	HeLD13	HeLD14
HeLD1	1.00													
HeLD2	.61^*^	1.00												
HeLD3	.35^*^	.34^*^	1.00											
HeLD4	.34^*^	.35^*^	.77^*^	1.00										
HeLD5	.37^*^	.37^*^	.41^*^	.42^*^	1.00									
HeLD6	.32^*^	.33^*^	.38^*^	.39^*^	.62^*^	1.00								
HeLD7	.32^*^	.36^*^	.43^*^	.41^*^	.46^*^	.38^*^	1.00							
HeLD8	.34^*^	.36^*^	.31^*^	.33^*^	.41^*^	.42^*^	.47^*^	1.00						
HeLD9	.37^*^	.39^*^	.39^*^	.39^*^	.55^*^	.55^*^	.47^*^	.46^*^	1.00					
HeLD10	.34^*^	.36^*^	.38^*^	.37^*^	.52^*^	.48^*^	.45^*^	.43^*^	.65^*^	1.00				
HeLD11	.35^*^	.35^*^	.39^*^	.37^*^	.44^*^	.40^*^	.43^*^	.36^*^	.46^*^	.47^*^	1.00			
HeLD12	.37^*^	.35^*^	.39^*^	.38^*^	.44^*^	.42^*^	.41^*^	.36^*^	.46^*^	.43^*^	.74^*^	1.00		
HeLD13	.30^*^	.34^*^	.39^*^	.36^*^	.45^*^	.37^*^	.34^*^	.31^*^	.39^*^	.39^*^	.45^*^	.44^*^	1.00	
HeLD14	.29^*^	.32^*^	.36^*^	.35^*^	.44^*^	.36^*^	.30^*^	.28^*^	.39^*^	.36^*^	.44^*^	.42^*^	.88^*^	1.00

^*^Significant correlation (*p* < 0.05)

#### Criterion validity

The A-HeLD-14 showed a statistically significant correlation with the overall oral rating (*r* = 0.37, *p* < 0.001).

#### Sensitivity analysis

The median A-HeLD-14 score for the entire population was 50.00 (IQR = 12.00). The median A-HeLD-14 score was 51.00 (IQR = 12.00) in the cities, 48.00 (IQR = 15.00) in the villages, and 47.00 (IQR = 19.00) in the hijrahs. The difference between all strata was statistically significant (*p* < 0.05). The median A-HeLD-14 score was higher in participants who brushed their teeth frequently, visited the dentist regularly, frequently consumed fresh fruit, and infrequently consumed soda or energy drinks compared to their counterparts in each variable. The differences were statistically significant (*p* < 0.05) (Table 4).

**Table 4:** Association between oral health-related behaviours and the A-HeLD-14 score, *n* = 1889

Oral health-related behaviours and the A-HeLD-14 score		
Variable	**Median A-HeLD-14 score (IQR)**	***P*-value**
Toothbrushing		
Frequent	51.31 (10.00)	< 0.01
Infrequent	41.50 (24.08)	
Dental attendance		
Regular	52.00 (12.00)	< 0.01
Irregular	49.00 (13.00)	
Fresh fruit		
Frequent	51.00 (12.00)	< 0.01
Infrequent	47.00 (14.00)	
Soda or energy drink		
Frequent	48.00 (15.00)	< 0.01
Infrequent	51.00 (11.00)	

**Fig. 2:**
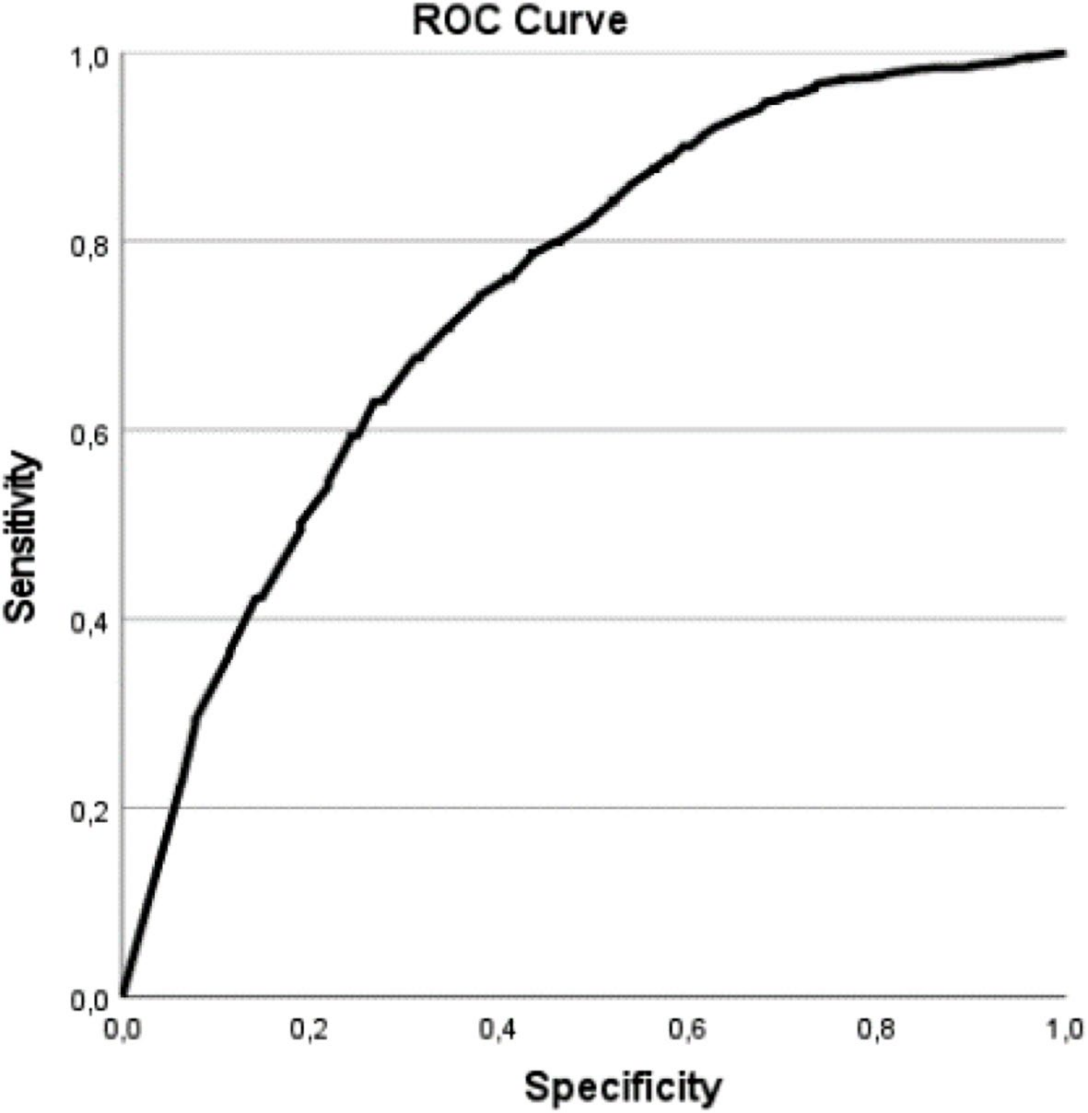
The ROC curve illustrating the relationship between the A-HeLD-14 and the frequency of toothbrushing

The ROC curve regarding frequency of toothbrushing is shown in Fig. 2. The AUC is 0.74 (*p* < 0.01), which indicates high sensitivity. The AUC for the other curves (not presented) is 0.6 (*p* < 0.01), which indicates poor discrimination.

## Discussion

This study evaluated the validity of the Arabic version of the HeLD-14 and found that the instrument showed good psychometric properties.

The test–retest reliability in this study was fair. This is in contrast with the results from the study evaluating the Brazilian version of HeLD-14, which found excellent test-retest reliability, with an ICC of 0.93 [16]. The relatively low ICC found in our study is difficult to explain as we complied with the standards of test–retest reliability. The data seem to indicate a ceiling effect as most participants had high A-HeLD-14 scores. This is natural since the population comprised younger women, but it limits the spread of the A-HeLD-14 score and thus could have affected the ICC. Further, the data showed that some participants had large discrepancies between test and retest, which is highly unlikely to be correct. One explanation could be that there were errors in answering due to careless responses or the nonoptimal arrangement of answer categories. Nevertheless, the A-HeLD-14 seems sufficiently reliable.

The content validity test showed that the high and moderate correlations, indicating an overlap between items of the A-HeLD-14, occurred mostly between items from the same domain. As the wording of the items within the domains is quite similar, this finding was expected and indicates the high content validity of the A-HeLD-14. The overlap between the receptivity and utilisation domains is interesting and unsurprising as taking actions towards good oral health is associated with the ability to invest time in and pay attention to oral health. The correlation between the items of a short version of HeLD was evaluated for the Brazilian version of HeLD-14 by assessing convergent and discriminant validity, revealing a strong interrelation between the items of each domain [16]. Criterion validity is another relevant aspect included in this study, since it helped in assessing how accurately the A-HeLD-14 measures oral health literacy. The correlation coefficients were satisfactory, although not high. This is compatible with the findings from another study that used the same approach to evaluate criterion validity and found a significant correlation between overall oral health and a Chinese version of the HeLD-14 of 0.26 [17].

The sensitivity tests indicated that higher OHL is associated with living in an urban area compared to rural areas, which is also in accordance with the findings of another study investigating OHL in relation to associated factors among undergraduate students in Malaysia [43]. High OHL was also strongly associated with frequent toothbrushing in the Mann–Whitney U test and in the ROC curve analysis. This is compatible with another study that found a significant correlation between frequent toothbrushing and oral health literacy among a group of students in Malaysia [29]. The association between low OHL and frequent soda and energy drink consumption was also assessed in other studies with findings similar to those of our study [28, 29, 44]. The sensitivity test also supported our hypothesis for a significant relationship between OHL and pattern of dental attendance. This is not surprising since many of the A-HeLD-14 items deal with dental attendance. Some other studies found a significant association between high OHL and regular dental attendance [28, 29, 45, 46], whereas another study found no significant association between OHL and the pattern of dental attendance [44]. Overall, the A-HeLD-14 is considered sensitive and the association with frequency of toothbrushing was found to be strong.

The internal consistency measured through Cronbach’s alpha is high and quite similar to the value found in the original validation study for the HeLD-14 [14] and in studies assessing the validity and reliability of the Brazilian and Chinese versions of the HeLD-14 [16, 17]. Moreover, the value is higher than the one found in the other study using an Arabic version of HeLD-14, which obtained a Cronbach’s alpha of 0.76 [20].

Other types of psychometric testing could have been relevant to include. In this regard, face validity would have helped to evaluate whether the questionnaire was suitable and presentable [47], and possibly explained the relatively low ICC. Additionally, more tests on the construct validity, including convergent validity, as well as concurrent and predictive validity when evaluating criterion validity would have been useful. Overall, our approach for validating the A-HeLD-14 is, however, considered comprehensive and appropriate, and the psychometric properties of the A-HeLD-14 considered sufficient, rendering the tool suitable for evaluating OHL among Arab populations.

### Strengths and limitations

This study has several strengths. The study sample was randomly collected from the entire target population in the Eastern Province of the KSA. No governorates were excluded and the sample can be considered representative of the target group and comprised participants of varying socio-economic status from urban and rural areas. These factors contribute to the generalisability of the findings. Furthermore, the sample for this study was considerably larger than that of other validation studies of the HeLD-14 with a response rate of nearly 100%, which helped to provide robust results. Collecting the sample via a school setting rather than healthcare services, hospitals or dental clinics enabled OHL to be assessed in a natural setting and thus avoided sampling bias.

This study also has limitations. The sample comprised only young females, which implies that generalisability of the study findings should be done with caution. Inclusion of other age groups and males in the study sample would in this regard have enhanced the generalisability of the study findings. This suggests that more validation studies in other populations are warranted, which is possible with the introduction of the A-HeLD-14. The A-HeLD-14 can also open the door to more studies being conducted on OHL in Arabic-speaking countries. In this regard, it is important to stress that although the HeLD-14 has been used to assess OHL among adolescents in different countries and in similar school settings [48, 49], the items of the HeLD-14 are not specifically aimed at an adolescent target population, which speaks for the usefulness of the tool in other populations.

## Conclusion

This validated A-HeLD-14 makes it possible for future epidemiological studies to assess OHL in Arab-speaking countries, helping to identify the barriers and facilitators of good oral health through OHL. Effective interventions could then be developed, implemented, and followed, which could have a positive impact on oral health states through better access to dental healthcare services and improved oral health-related behaviours as well as decision making. The results of this study are thus relevant for governments and health authorities in Arabic-speaking countries.

The A-HeLD-14 is a valid, reliable, and sensitive instrument for measuring oral health literacy.

## Data Availability

The datasets generated and analysed during the current study are not publicly available due to the confidentiality of research data but are available from the corresponding author on reasonable request.

## Abbreviations

A-HeLD-14: The Arabic version of the short version of the Health Literacy in Dentistry scale
AUC: Area Under Curve
HeLD-14: The Health Literacy in Dentistry scale
ICC: Intra-class Correlation Coefficient
IQR: Interquartile Range
KSA: The Kingdom of Saudi Arabia
OHL: Oral Health Literacy
REALD: Rapid Estimate of Adult Literacy in Dentistry
ROC: Receiver Operating Characteristic (curve)
WHO: The World Health Organisation
